# Pellet coculture of osteoarthritic chondrocytes and infrapatellar fat pad-derived mesenchymal stem cells with chitosan/hyaluronic acid nanoparticles promotes chondrogenic differentiation

**DOI:** 10.1186/s13287-017-0719-7

**Published:** 2017-11-15

**Authors:** Shu Huang, Xiongbo Song, Tao Li, Jingfang Xiao, Yemiao Chen, Xiaoyuan Gong, Weinan Zeng, Liu Yang, Cheng Chen

**Affiliations:** 1 Center for Joint Surgery, Southwest Hospital, Third Military Medical University, Chongqing, China; 2 Institute of Pathology and Southwest Cancer Center, Southwest Hospital and Key Laboratory of Tumor Immunopathology of the Ministry of Education of China, Third Military Medical University, Chongqing, China

**Keywords:** Msenchymal stem cells, Chondrocytes, Chondrogenesis, Osteoarthritis

## Abstract

**Background:**

Cell source plays a key role in cell-based cartilage repair and regeneration. Recent efforts in cell coculture have attempted to combine the advantages and negate the drawbacks of the constituent cell types. The aim of this study was to evaluate the chondrogenic outcome of articular chondrocytes (ACs) and infrapatellar fat pad (IPFP)-derived mesenchymal stem cells (MSCs) in direct coculture.

**Methods:**

ACs and IPFP MSCs from the same patients with knee osteoarthritis (OA) were cocultured in monolayer and in pellets. The monocultures of each cell type were also used as controls. Morphological and histologic analysis, immunofluorescence staining, reverse transcription-polymerase chain reaction, and enzyme-linked immunosorbent assay were performed to characterize the chondrogenic differentiation of cocultures. Furthermore, the effects of chitosan/hyaluronic acid (CS/HA) nanoparticle exposure on the chondrogenesis of cocultures were examined.

**Results:**

In both monolayer and pellet coculture, the hypertrophy of MSCs and the inflammatory activities of ACs were inhibited, although the chondrogenic production in coculture was not promoted compared with that in monoculture. In addition, the exposure of CS/HA nanoparticles to pellet coculture improved the production of type II collagen and aggrecan.

**Conclusions:**

We demonstrate for the first time that pellet coculture of ACs and IPFP MSCs with CS/HA nanoparticles could promote chondrogenic outcome while preventing the inflammatory status of ACs and the hypertrophic differentiation of MSCs. These findings suggest that the combination of ACs, IPFP MSCs, and CS/HA might be useful in cartilage repair in knee OA.

## Background

Osteoarthritis (OA) is the most prevalent form of joint disease [1], and is the leading global cause of disability [2]. A common hallmark of OA is the progressive loss of articular cartilage due to its avascular nature and low mitotic activity of articular chondrocytes (ACs) embedded within the dense cartilage extracellular matrix (ECM) [3]. As such, significant research efforts are aimed at producing engineered cartilage as a cell-based approach for articular cartilage repair [4–6]. The most commonly used cell sources in cartilage tissue engineering and regenerative medicine are native cartilage cells, or autologous chondrocytes, but they undergo rapid dedifferentiation during in-vitro expansion, and result in the formation of fibrocartilage with inferior mechanical properties [7, 8]. Mesenchymal stem cells (MSCs) have been widely investigated as an alternative cell source in cartilage regeneration due to their great chondrogenic potential [9, 10]. However, MSCs with the current chondrogenic differentiation protocol usually result in the hypertrophic phenotype and calcification [11, 12].

To avoid the need for autologous chondrocyte expansion, and to enhance MSC differentiation to the chondrogenic lineage, the coculture approach [13, 14] of both cell types has been developed recently and has shown promising results. By coculturing rabbit chondrocytes with MSCs, Shi et al. found that the proliferation of chondrocytes is promoted and the chondrogenic differentiation of MSCs is enhanced [15]; Xu et al. further confirmed that the proliferation of rabbit chondrocytes is provoked by the paracrine factors of MSCs [16]. Studies with human cells have also been encouraging; for example, Li et al. showed that human umbilical cord blood-derived MSCs contribute to chondrogenesis in coculture with chondrocytes [17], and Zhang et al. cocultured human osteoarthritic chondrocytes with bone marrow-derived MSCs and found that the proliferation of chondrocytes is increased and the inflammatory activity of chondrocytes is inhibited [18]. Although these intensive research efforts have generated positive findings concerning coculture of MSCs and ACs, the clinical translation of the coculture approach remains challenging. First, it requires extra invasive procedures to isolate MSCs from other tissues, which increases pain and cost for the patients; second, the success of some in-vitro coculture studies relies on the indirect contact of MSCs and ACs, which is difficult to implement in vivo.

Here, we seek to develop a new strategy that avoids not only the isolation of normal chondrocytes from non-load bearing areas, but also the extra procedure for obtaining MSCs from other tissues. Following this idea, we cocultured human infrapatellar fat pad (IPFP)-derived MSCs and osteoarthritic ACs since both cells could be obtained from a single arthroscopy. Our data demonstrated that pellet coculture in vitro inhibits both the hypertrophy of IPFP MSCs and the inflammatory activity of osteoarthritic chondrocytes. Moreover, the chondrogenesis of cells in pellet coculture is promoted in the presence of chitosan/hyaluronic acid (CS/HA) nanoparticles (NPs). Results of this study indicate the possibility of developing a one-step cartilage repair by using a combination of IPFP MSCs, ACs, and CS/HA NPs for patients with knee OA.

## Methods

Human IPFPs and cartilage tissues were obtained intraoperatively from total knee arthroplasties after informed consent and approval from the Ethics Committee of Southwest Hospital (Chongqing, China). Only patients with primary knee OA (grade IV in The Kellgren Lawrence grading system) were selected, and patients with inflammatory arthritis or with a history of prior knee surgery were excluded.

### Cell isolation and characterization

Donor-matched infrapatellar fat pad and articular cartilage were obtained from four OA patients (two male and two female, average age 65 ± 3 years). For isolation of IPFP MSCs, infrapatellar fat pad was harvested, washed in phosphate-buffered saline (PBS) supplemented with 1% penicillin/streptomycin (P/S; Beyotime), and then diced into 2-mm pieces. The diced tissues were digested in 0.1% type I collagenase (Sigma) for 2 h, and the resulting cell suspension was filtered through a 40-μm cell strainer (BD Bioscience). The collected cells were centrifuged (400 g for 5 min) and resuspended in Red Cell Lysis Buffer (Beyotime) at room temperature in the dark for 10 min. The cells were then centrifuged again, and resuspended in Dulbecco’s modified Eagle’s medium (DMEM)/F12 (Invitrogen) supplemented with 10% fetal bovine serum (FBS; Gibco) and 1% P/S. The medium was changed every 2 days.

For isolation of ACs, cartilage specimens were washed in PBS supplemented with 1% P/S and then diced. ACs from the diced tissues were isolated by digesting the matrix overnight in high-glucose DMEM (Invitrogen) supplemented with 0.2% type II collagenase (Sigma) and 1% P/S. The resulting cell suspension was filtered through a 40-μm cell strainer; collected cells were centrifuged (400 g for 5 min), and resuspended in high-glucose DMEM supplemented with 10% FBS and 1% P/S. The medium was changed every 2 days.

IPFP MSCs were characterized using flow cytometry and the Human MSC Analysis Kit (BD Biosciences). Briefly, cells were resuspended at a concentration of 1 × 10^7^ cells/ml in 0.5% bovine serum albumin in PBS. Cells aliquots (100 μl per tube) were stained with mesenchymal cell markers (CD90, CD105, CD73, and CD44) and immunoglobulin (Ig)G1 and IgG2a isotype controls.

### Cell coculture and cell labeling

IPFP MSCs at passage 2 and ACs at passage 0 from a single patient were used in coculture. For monolayer coculture, IPFP MSCs (2 × 10^5^ cells) and ACs (2 × 10^5^ cells) in 2 ml chondrogenic medium were seeded onto each well of a six-well plate. IPFP MSCs alone (4 × 10^5^ cells per well) and ACs alone (4 × 10^5^ cells per well) were used as controls. For three-dimensional pellet coculture, a cell suspension containing 1 × 10^6^ IPFP MSCs and 1 × 10^6^ ACs was centrifuged at 1000 rpm for 5 min in 15-ml polypropylene conical tubes. The supernatant was then removed, and 2 ml chondrogenic medium was gently added into each tube without disturbing the cell sediment. IPFP MSCs alone (2 × 10^6^ cells per tube) and ACs alone (2 × 10^6^ cells per tube) were used as controls. For both two- and three-dimensional coculture, the chondrogenic medium used was Advanced DMEM (Gibco) supplemented with 1% P/S, 40 μg/ml l-proline (Biosharp), 50 μg/ml insulin-transferrin-selenium-A supplement (Gibco), 10 ng/ml recombinant human transforming growth factor (TGF)-β3 (Peprotech), 0.1 mM ascorbic acid 2-phosphate (Sigma), and 100 nM dexamethasone (Sigma). Cells were maintained at 37 °C and 5% CO_2_, and the chondrogenic medium was replaced every 3 days.

To distinguish these two cell types and to evaluate cell survival during coculture, IPFP MSCs and ACs were incubated with the fluorescent dyes 1,1'-dioctadecyl-3,3,3',3'-tetramethylindocarbocyanine perchlorate (DiI; 10 μM, Beyotime) and 3,3′-dioctadecyloxacarbocyanine perchlorate (Dio; 10 μM, Beyotime), respectively, before coculture. After 1 week of coculture, fluorescent images of the cells were taken and the cells were counted.

### Hematoxylin and eosin staining and Alcian blue staining

After 3 weeks of coculture, hematoxylin and eosin (H&E) staining for cell and matrix distribution and Alcian blue staining for proteoglycan were performed. For H&E staining, a staining kit (Beyotime) was used. For Alcian blue staining, samples were equilibrated in 3% glacial acetic acid for 30 min, stained with 0.1% Alcian blue (Biosharp) dissolved in 3% glacial acetic acid (pH 2.5) for 30 min with constant agitation, and rinsed with 3% glacial acetic acid three times for 30 min each.

### Immunofluorescence staining

After 3 weeks of coculture, cells in the monolayer were fixed in 4% paraformaldehyde, and cells in pellet were frozen-sectioned. Non-specific bindings were blocked with 1% bovine serum albumin in PBS for 60 min. Fixed cells were incubated with primary antibody (COL I, ab6038; COL II, ab185430; Aggrecan,ab3778; SOX9, ab185230; 1:100; Abcam) overnight at 4 °C, followed by incubation with secondary antibody (Ms-488 or Rb-488; ZSGB-BIO) for 60 min at room temperature. Nuclei were stained with 0.1 μg/ml 4’6-diamidino-2-phenylindole (DAPI; Sigma) in PBS for 1 min. Immunostained samples were observed by an Olympus IX71 microscope.

### Reverse transcription-polymerase chain reaction (RT-PCR)

RT-PCR was used to analyze the gene expression of chondrogenic markers. Total RNA was isolated from the cell monolayer or pellets using TRIzol (Takara) following the manufacture’s protocol, and a RevertAid First Strand cDNA Synthesis Kit (Thermo Fisher Scientific). The target gene primers were designed as follows: *COL1A1*, forward, CCTGGATGCCATCAAAGTCT, reverse, AATCCATCGGTCATGCTCTC; *COL2A1*, forward, TGCTGCCCAGATGGCTGGAGGA, reverse, TGCCTTGAAATCCTTGAGGCCC; *COL10A1*, forward, GGGAGTGCCATCATCG, reverse, GAGGCTTCACATACGTTT; *ACAN*, forward, TCGAGGACAGCGAGGCC, reverse, TCGAGGGTGTAGCGTGTAGAGA; *SOX9*, forward, GACTTCCGCGACGTGGAC, reverse, GTTGGGCGGCAGGTACTG; GAPDH, forward, GAGAACGGGAAACTTGTCAT, reverse, GGCAGGTCAGGTCAACAA. Real-time PCR was performed using the PCR kit PowerUp SYBR Green Master Mix (Thermo Fisher Scientific). Assays were performed in triplicate.

### Enzyme-linked immunosorbent assay (ELISA)

The supernatant of the culture medium was collected at days 7, 14, and 21 of coculture. The expression of metallopeptidase (MMP)-13 (CSB-E04674h), interleukin (IL)-1β (CSB-E08053h), and a disintegrin and metalloproteinase with thrombospondin motifs (ADAMTS)5 (CSB-EL001312HU) were detected using ELISA (CUSABIO). According to the kit instructions, 100 μl standard or sample was added to each well and incubated for 2 h at 37 °C. Then 100 μl Biotin antibody (1×) was added to each well and incubated for 1 h at 37 °C. After three washes, 100 μl HRP-avidin (1×) was added to each well and incubated for 1 h at 37 °C. After five washes, 90 μl TMB Substrate was added to each well and incubated for 20 min at 37 °C in the dark. Finally, 50 μl Stop Solution was added to each well. The optical density (OD) value of each well was measured at a wavelength of 450 nm. This was positively correlated to its respective concentration of MMP-13, IL-1β, and ADAMTS 5. The experiment was repeated three times.

### Fabrication and characterization of CS/HA NPs

CS/HA NPs were prepared as previously described with minor modification [19, 20]. Briefly, CS (100 kDa) was dissolved in 2% acetic acid at a concentration of 1% (w/v), and HA (10 kDa) was dissolved in water at a concentration of 0.1% (w/v). Both CS and HA solutions were mixed at a ratio of 1:1 (v/v) and sonicated for 15 min at 25 °C to form NPs. Eight different mixtures were prepared with CS/HA weight ratios at 1:2, 1:1, 2:1, 3:1, 4:1, 5:1, 6:1, and 7:1. Accordingly, a constant HA concentration of 50 μg/ml was used in all mixtures, and the CS concentration was varied as 25, 50, 100, 150, 200, 250, 300, and 350 μg/ml.

To measure the size and charge of the CS/HA NPs, dynamic light scattering and zeta-potential measurement of NPs in aqueous solution were performed with the Malvern Zetasizer Nano ZS instrument (Malvern, UK) at 25 °C. The samples were dried at room temperature after being stained on copper grids, and then transmission electron microscopy (TEM) was performed on a TECNAI-10 microscope (Philips) at an acceleration voltage of 50 kV to observe the particle morphology. FITC-conjugated CS/HA NPs were used to confirm the cellular intake of these NPs.

### Statistical analysis

Data were expressed as mean ± standard deviation (SD). Statistically significant differences between test groups were determined by one-way analysis of variance (ANOVA) at a confidence interval of 95%. In addition, pairwise comparisons were made after ANOVA using the Holm-Sidak test. SigmaStat (Systat Software) was used for performing all the statistical analysis.

## Results

### Cell characterization

**Fig. 1:**
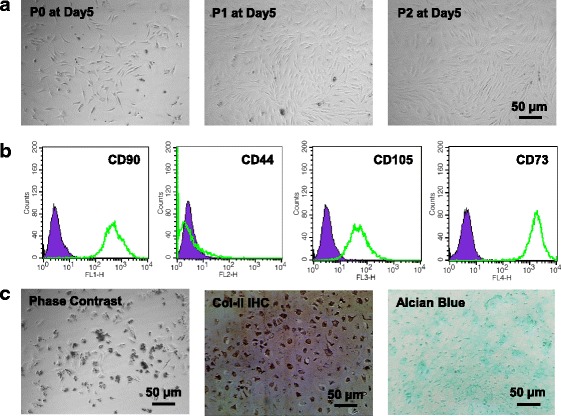
Characterization of infrapatellar fat pad-derived mesenchymal stem cells (MSCs) and articular chondrocytes (ACs). **a** Phase contrast images of P0, P1, and P2 MSCs. **b** MSCs were tested for mesenchymal surface markers (CD90, CD105, and CD73) by flow cytometry. **c** Phase contrast image, immunohistochemistry (*IHC*) of type II collagen (*Col II*), and Alcian blue staining of P0 ACs

The isolated IPFP MSCs based on plastic adhesion displayed a spindle-shaped morphology typical of MSCs (Fig. 1a). Flow cytometry analysis (Fig. 1b) showed that these cells were positive for the surface markers CD90 (88.39%), CD105 (83.43%), and CD73 (88.43%). Although expressing weakly for CD44 (4.32%), these cells conformed to the minimal identification of human MSCs proposed by the International Society for Cellular Therapy [21].

The primary ACs had a polygon, spindle, or irregular shape (Fig. 1c, left). After immunolocalization of type II collagen, brown-yellow granules were found in the cells (Fig. 1c, middle). Alcian blue staining revealed that the chondrocyte cytoplasm and matrix were stained greenish-blue (Fig. 1c, right), indicating the deposition of glycosaminoglycans (GAGs).

### Preliminary observation of coculture

**Fig. 2:**
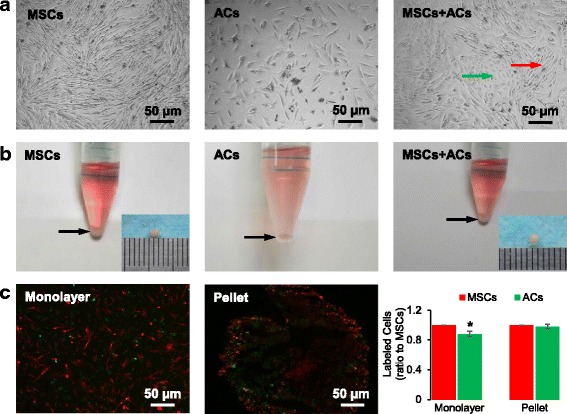
Cell morphology and cell labeling after 1 week of chondrogenic coculture. **a** Phase contrast images of IPFP mesenchymal stem cells (*MSCs*) (*left*), articular chondrocytes (*ACs*) (*middle*), and coculture of MSCs and ACs (*right*) in monolayer. The *green arrow* points to ACs, and the *red arrow* points to MSCs in coculture. **b** Macroscopic observation of MSCs (*left*), ACs (*middle*), and coculture of MSCs and ACs (*right*) in pellets. *Arrows* point to cell sediments after 1 week of culture; inserts illustrate the size of the pellets. **c** MSCs were labeled with DiI (*red*) and ACs were labeled with Dio (*green*) in monolayer (*left*) and pellet (*middle*) coculture. After 1 week of coculture, the labeled cells were counted, and the ratio of AC number to MSC number was calculated (right). Data are presented as mean ± SD. *P < 0.05

After 1 week of coculture in chondrogenic medium, we observed the cell morphology and viability. In monolayer culture, the morphology of both IPFP MSCs (Fig. 2a, left) and ACs (Fig. 2a, middle) did not change compared with cells in the growth medium; in the coculture image (Fig. 2a, right), most ACs could be distinguished from IPFP MSCs. In pellet culture (Fig. 2b), cell deposition was clearly observed at the bottom of the 15-ml tube. After being taken out of the tube, the pellets from the IPFP MSC group and coculture group were both about 2 mm in size (Fig. 2b, inserts), while the pellet from the AC group collapsed due to inadequate strength. The number of labeled cells in the monolayer (Fig. 2c, left) or in the pellet (Fig. 2c, middle) was calculated using ImageJ (Fig. 2c, right); in monolayer coculture, the ratio of AC number to IPFP MSC number was 0.88 ± 0.04, indicating more loss of ACs; in pellet coculture, the ratio of AC number to IPFP MSC number was close to 1, indicating a balanced viability of both cells.

### Histologic analysis and immunofluorescence staining

**Fig. 3:**
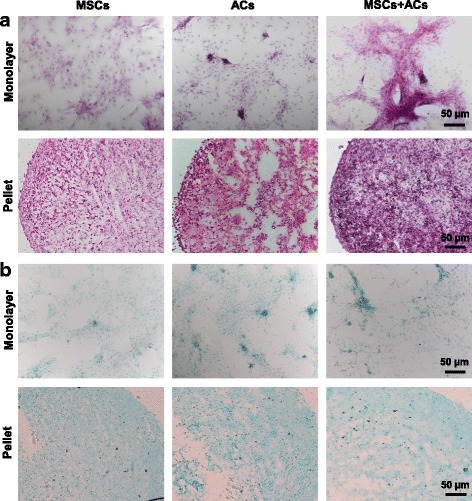
Histological analysis of monoculture of IPFP mesenchymal stem cells (*MSCs*), monoculture of articular chondrocytes (*ACs*), and coculture of MSCs and ACs after 3 weeks of chondrogenic induction. **a** H&E staining showing cell and matrix distribution. **b** Alcian blue staining showing GAG deposition

After 3 weeks of coculture, H&E staining and Alcian blue staining were performed. In general, cells in pellets showed a better matrix deposition and GAG production compared to cells in monolayer. Specifically, for H&E staining (Fig. 3a), cells in the monolayer coculture group formed a large cell cluster; cells in the pellet coculture group formed a denser pellet compared to pellet culture of IPFP MSCs or ACs alone. In particular, for Alcian blue staining (Fig. 3b), the coculture group did not show much difference compared with the other groups in either monolayer or pellet culture.

**Fig. 4:**
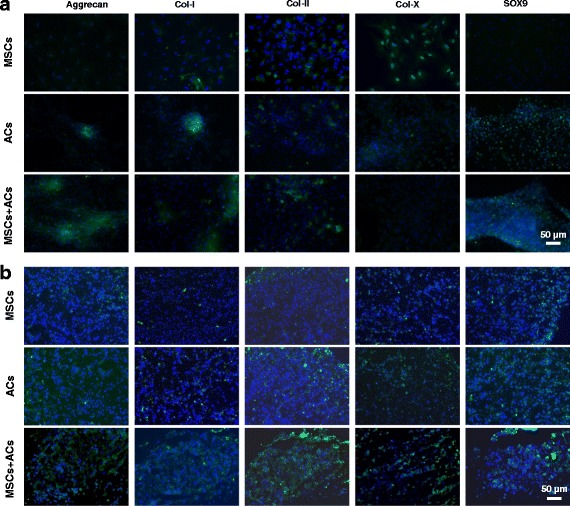
Immunofluorescence assay of monoculture of IPFP mesenchymal stem cells (*MSCs*), monoculture of articular chondrocytes (*ACs*), and coculture of MSCs and ACs after 3 weeks of chondrogenic induction. **a** Fluorescent images of two-dimensional monolayers. **b** Fluorescent images of three-dimensional pellet sections. *Col* collagen

The immunofluorescence of SOX9 and major ECM proteins (type I, II, and X collagen) were also detected. In monolayer culture (Fig. 4a), the IPFP MSC group was abundant in type X collagen, the AC group was abundant in type I collagen, and the coculture group was abundant in both type I and type II collagen. Moreover, the staining of the chondrogenic markers aggrecan and SOX9 in the coculture group was much stronger than that in the IPFP MSC group or the AC group. In pellet sections (Fig. 4b), the staining of both type I and type II collagen was stronger in the coculture group than that in the IPFP MSC group or the AC group, while the staining of aggrecan and SOX9 was relatively similar among the groups.

### Effects of CS/HA on the chondrogenesis of coculture

**Fig. 5:**
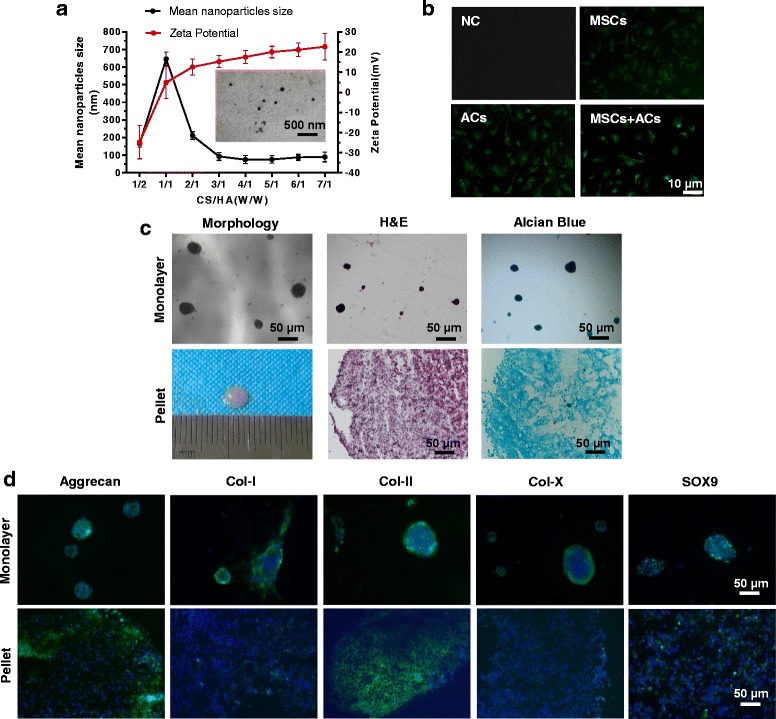
Effects of chitosan/hyaluronic acid (*CS/HA*) NP exposure on IPFP mesenchymal stem cell (*MSC*) and articular chondrocyte (*AC*) cocultures. **a** Effect of the weight ratio of CS to HA on the size and zeta potential of NPs. Insert: TEM micrograph of CS/HA NPs. **b** Fluorescent images of cells exposed to FITC-conjugated CS/HA NPs for 1 h. **c** Morphology, hematoxylin and eosin (*H&E*), and Alcian blue staining of monolayer (*top*) and pellet (*bottom*) culture of MSCs + ACs + NPs. **d** Immunofluorescence assay of monolayer (*top*) and pellet (*bottom*) culture of MSCs + ACs + NPs. *Col* collagen, *NC* negative control

The relationship between the weight ratio of CS/HA and the size of the NPs is shown in Fig. 5a. There was a significant increase in nanoparticle size when the CS/HA ratio increased from 1:2 to 1:1; with the increasing amount of CS, the size of the NPs decreased to 100 nm less. The smallest particle size (74.6 ± 7.6 nm) was obtained at the CS/HA weight ratio of 4:1. The average zeta potential became more positive with the increase in the CS amount within the polyelectrolyte complex. TEM image (Fig. 5a, insert) showed that the CS/HA NPs were spherical in shape and well dispersed. For subsequent experiments, CS/HA NPs with a CS/HA weight ratio of 4:1 were used. The fluorescent images (Fig. 5b) indicated that many FITC-conjugated NPs were taken by the cells.

After 3 weeks of exposure to HA/CS NPs, the morphology and histology of the coculture group was examined. In monolayer coculture (Fig. 5c, top), the cells lost adherence to the culture dish and formed spheres. In pellet coculture (Fig. 5c, bottom), the pellet was about 4 mm in size; H&E staining showed that the coculture + NP group was comparable in cell density to the pellet coculture group, while Alcian blue staining indicated a higher production of GAGs after NP intake.

Fluorescent staining of the marker proteins was also performed. In monolayer coculture (Fig. 5d, top), the intensity and distribution of the marker proteins could not be determined because the cells formed spheres; in pellet coculture (Fig. 5d, bottom), fluorescent staining indicated a higher expression of aggrecan and type II collagen in the presence of HA/CS NPs (compared with the lower-right images in Fig. 3a and b).

### Quantitative analysis of chondrogenic markers and inflammatory markers

**Fig. 6:**
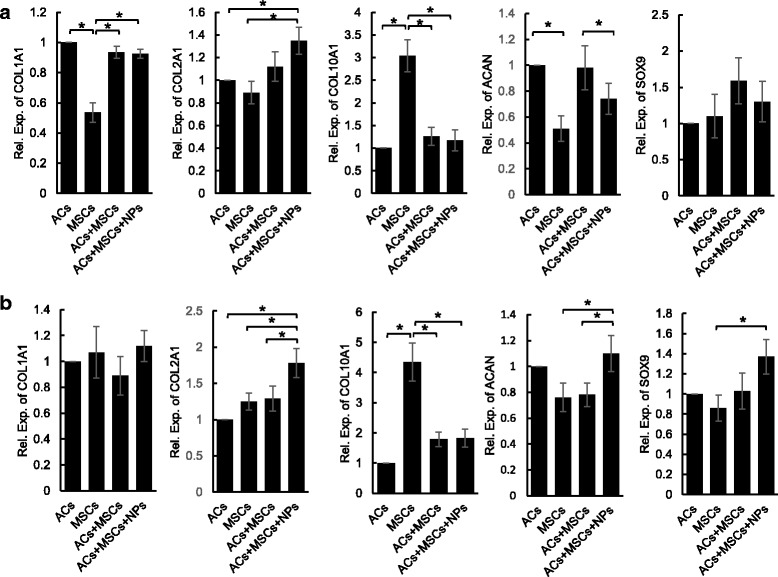
Expression profile of COL1A1, COL2A1, COL10A1, ACAN, and SOX9 after 3 weeks of chondrogenic induction in monolayer (**a**) and pellet (**b**) culture. Gene expression of each group was normalized to the AC group. Data are presented as mean ± SD. *P < 0.05. *AC* articular chondrocyte, *MSC* mesenchymal stem cell, *NP* nanoparticle

The mRNA expression of the chondrogenic markers *COL2A1*, *SOX9*, and *ACAN*, the fibrotic marker *COL1A1*, and the hypertrophic marker *COL10A1* in both monolayer and pellet culture was analyzed after 3 weeks of culture. In monolayer culture (Fig. 6a), the MSC group had significantly lower expression of *COL1A1* but significantly higher expression of *COL10A1* than the other groups, indicating that two-dimensional coculture did not weaken the fibrosis of chondrocytes but could inhibit the hypertrophy of IPFP MSCs. Furthermore, the coculture + NP group had higher *COL2A1* expression than the AC group or the MSC group alone, and lower *ACAN* expression than the coculture group. In pellet culture (Fig. 6b), the coculture + NP group had significantly higher *COL2A1* expression than the other groups, and higher *ACAN* expression than the coculture group or the MSC group, suggesting that the addition of NPs greatly improves the production of type II collagen and aggrecan in pellet coculture.

**Fig. 7:**
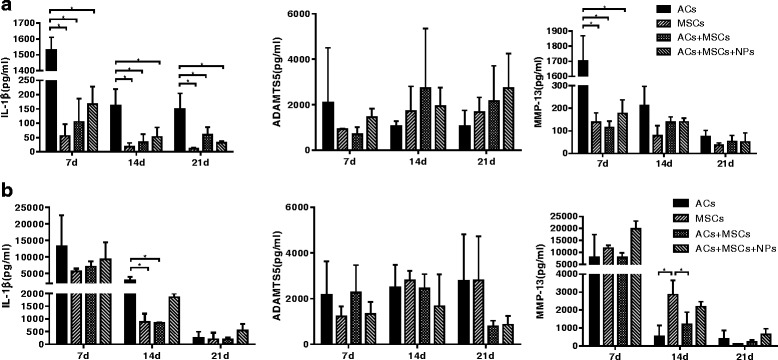
Concentration of interleukin-1β (*IL-1β*), a disintegrin and metalloproteinase with thrombospondin motifs 5 (*ADAMTS-5*), and metallopeptidase 13 (*MMP-13*) at 1 week, 2 weeks, and 3 weeks of chondrogenic induction in monolayer (**a**) and pellet (**b**) culture. Data are presented as mean ± SD. *P < 0.05. *AC* articular chondrocyte, *MSC* mesenchymal stem cell, *NP* nanoparticle

To determine the level of cytokines and enzymes (IL-1β, ADAMTS5, and MMP-13) during coculture, the medium at 1 week, 2 weeks, and 3 weeks was collected and analyzed using ELISA. In general, there was a downward trend in concentration of IL-1β and MMP-13 as culture time increased in both monolayer culture and pellet culture. In monolayer culture (Fig. 7a), the AC group kept expressing a higher level of IL-1β than the other groups, and only expressed higher expression of MMP-13 than the other group in the first week. In pellet culture (Fig. 7b), a significant difference was only detected at 2 weeks, and there was no difference of cytokine concentration among the groups at 1 week or at 3 weeks. Note that there was no difference in cytokine/enzyme concentration between the coculture group and the coculture + NP group, indicating that the presence of NPs neither stimulated nor restrained the release of these cytokine/enzymes.

## Discussion

The success of cell-based therapies for cartilage-related injury and disease requires fine control of cell differentiation. However, chondrocytes alone usually end up showing the fibrotic phenotype, while MSCs alone lead to the hypertrophic phenotype in cartilage tissue engineering. For this reason, chondrocyte-MSC coculture systems have been recently employed in several studies [22] to limit chondrocyte dedifferentiation and MSC hypertrophy. The tissue sources of MSCs used in those studies are distinct from articular cartilage; thus, multiple procedures are needed to isolate chondrocytes and MSCs for coculture. In this study, we selected human IPFP MSCs to coculture with osteoarthritic chondrocytes. The biggest advantage of IPFP MSCs over other stem cells is that IPFP MSCs along with chondrocytes can be extracted from the same knee joint during a single knee arthroscopy, thereby avoiding multiple invasive procedures. The safety and suitability of IPFP MSCs from OA patients has also been proven in vitro [23–25] and in clinical studies [26, 27]. Furthermore, it has been shown that IPFP MSCs have a higher capacity for chondrogenic differentiation than MSCs from body fat, bone marrow, and Wharton’s jelly of the umbilical cord [28].

Here, we cocultured IPFP MSCs and ACs from the same osteoarthritic knee in both monolayer and pellet culture. We did not examine the effects of cell ratio in this study, but instead simply chose a 50:50 ratio of MSCs to ACs since it has been shown that this ratio provides optimal type II collagen expression and GAG production in several studies [29–31]. However, the maximum ratio of MSCs to ACs still needs investigation in further studies. In terms of culture condition, the pellet culture, a well-established three-dimensional culture method without scaffolding materials, exhibited more matrix deposition compared with monolayer culture, as evidenced by our H&E and Alcian blue staining results. In particular, when we cocultured ACs with IPFP MSCs in monolayer in chondrogenic medium for 1 week, there was a significant loss of chondrocyte numbers. This result contradicts a previous study [32] in which the human chondrocytes had a fourfold increase in cell numbers after 7 days of monolayer culture in chondrogenic medium. Despite this discrepancy, our major finding and that of the other study both exhibit the advantage of three-dimensional culture for chondrogenesis.

Interestingly, our PCR analysis revealed that, whether in monolayer or pellet culture, there was no significant difference in expression of the marker genes *COL2A1*, *ACAN*, and *SOX9* between the coculture group and the monoculture control, indicating that coculture did not change the chondrogenic capacity of both cells. In addition, there was no significant difference in *COL1A1* expression between the AC monoculture group and the coculture group, indicating that the fibroblastic differentiation of ACs was not reduced in coculture; however, there was a significant difference in *COL10A1* expression between the MSC monoculture group and the coculture group, indicating that hypertrophy of MSCs was inhibited in coculture. This inhibition of MSC hypertrophy might be attributed to the parathyroid hormone-related protein (PTHrP) secreted by ACs throughout the coculture [33, 34].

We further added CS/HA NPs into the AC-MSC coculture. Both CS and HA have been widely used in clinics; in particular, HA injection has been used as a treatment for knee OA [35]. Moreover, the CS/HA NPs are usually used as drug delivery vehicles [36, 37]. Therefore, the use of CS/HA NPs might add beneficial effects to coculture, and would facilitate drug loading (such as growth factors and anti-inflammatories) if necessary. In monolayer coculture, the cells lost adherence to the culture dish and formed spheres after the addition of CS/HA NPs. Similarly, in another study, MSCs formed spheres after transwell coculture with chondrocytes in the presence of TGF-β [38]. Therefore, we speculate that, given strong chondrogenic stimuli (HA, TGF-β, and so forth), MSCs in monolayer culture favor cellular aggregation rather than plastic adhesion. In pellet coculture, the addition of CS/HA NPs increased the expression level of the marker genes *COL2A1* and *ACAN*, indicating that the chondrogenesis of pellet coculture was promoted by CS/HA NPs. This could be attributed to the presence of HA, since it has been shown that HA causes a fourfold increase in chondrogenic differentiation of IPFP MSCs [28].

Since the ACs and IPFP MSCs were both from OA patients, the inflammatory state of the cells should be a concern. Here, we examined the concentration of IL-1β (a major cytokine in OA [39]), ADAMTS 5 (an enzyme responsible for aggrecan degradation [40]), and MMP-13 (an enzyme responsible for collagen degradation [41]) during coculture. We found that the concentration of both IL-1β and MMP-13 was highest in monoculture of ACs as expected since P0 osteoarthritic chondrocytes without passaging were used. Interestingly, the concentration of IL-1β and MMP-13 was comparable between the MSC group and the coculture group, suggesting an anti-inflammatory influence of MSCs on ACs. This phenomenon is possibly because MSCs could exert an anti-inflammatory effect on osteoarthritic chondrocytes through prostaglandin E_2_ [42] or MSC-secreted insulin-like growth factor 1 [43]. During coculture, a gradual decline in concentration of IL-1β and MMP-13 was observed, which was possibly due to repetitive medium changes that diluted the soluble inflammatory factors.

The major findings of this study were as follows. First, in coculture the hypertrophic differentiation of IPFP MSCs was weakened and the inflammatory state of ACs was alleviated, although the chondrogenic capacity of both cells did not change compared with cells in monoculture. Second, in pellet coculture, the presence of CS/HA NPs greatly improved the chondrogenic outcome. Based on these findings, we propose the use of the combination of autologous IPFP MSCs, osteoarthritic chondrocytes, and CS/HA NPs for treating knee OA. In fact, several attempts have been made using the combination of ACs and MSCs for chondrogenesis. Lopez-Ruiz et al. demonstrated that extracts obtained from chondrocytes of osteoarthritic knees could promote the chondrogenic differentiation of IPFP MSCs [44]. Arora et al. cocultured goat IPFP MSCs and ACs with plasma clots for up to 28 days, and observed the formation of hyaline cartilage-like matrix. Hence they suggested the potential application of cocultured IPFP MSCs and chondrocytes in cartilage tissue engineering [45]. Notably, Lopa et al. reported that osteoarthritic chondrocytes failed to induce chondrogenesis of donor-matched IPFP MSCs during pellet coculture; thus they suggested that specific attention should be given to donor age and OA grade [46]. In our study, we for the first time report the positive chondrogenic outcome of pellet coculture using CS/HA NPs in combination with ACs and IPFP MSCs from OA patients. However, there are two limitations that need our further investigation. First, the influence of cell ratio, donor age, and OA grade on the chondrogenic outcome of IPFP MSC-AC cocultures was not included; second, mechanisms of the attenuated AC inflammation and the inhibited MSC hypertrophy were not determined.

## Conclusions

In conclusion, our results reveal that the benefit of AC-IPFP MSC coculture is the inhibition of AC inflammation and prevention of MSC hypertrophy. Moreover, we showed here that the chondrogenic outcome of pellet coculture was improved when cells were exposed to CS/HA NPs, which was possibly due to the presence of HA. These findings suggest the potential of the AC-IPFP MSC-HA combination for cartilage repair in patients with knee OA.

## Abbreviations

AC: Articular chondrocyte
ADAMTS: A disintegrin and metalloproteinase with thrombospondin motifs
ANOVA: Analysis of variance
CS: Chitosan
DMEM: Dulbecco’s modified Eagle’s medium
ECM: Extracellular matrix
ELISA: Enzyme-linked immunosorbent assay
FBS: Fetal bovine serum
GAG: Glycosaminoglycan
H&E: Hematoxylin and eosin
HA: Hyaluronic acid
Ig: Immunoglobulin
IL: Interleukin
IPFP: Infrapatellar fat pad
MMP: Metallopeptidase
MSC: Mesenchymal stem cell
NP: Nanoparticle
OA: Osteoarthritis
P/S: Penicillin/streptomycin
PBS: Phosphate-buffered saline
PTHrP: Parathyroid hormone-related protein
RT-PCR: Reverse transcription-polymerase chain reaction
TEM: Transmission electron microscopy
TGF: Transforming growth factor
